# Molecular cytogenetic characterization of repetitive sequences comprising centromeric heterochromatin in three Anseriformes species

**DOI:** 10.1371/journal.pone.0214028

**Published:** 2019-03-26

**Authors:** Yoshinobu Uno, Chizuko Nishida, Ayano Hata, Satoshi Ishishita, Yoichi Matsuda

**Affiliations:** 1 Laboratory of Avian Bioscience, Department of Animal Sciences, Graduate School of Bioagricultural Sciences, Nagoya University, Nagoya, Aichi, Japan; 2 Department of Natural History Sciences, Faculty of Science, Hokkaido University, Sapporo, Hokkaido, Japan; 3 Avian Bioscience Research Center, Graduate School of Bioagricultural Sciences, Nagoya University, Nagoya, Aichi, Japan; University of Florence, ITALY

## Abstract

The highly repetitive DNA sequence of centromeric heterochromatin is an effective molecular cytogenetic marker for investigating genomic compartmentalization between macrochromosomes and microchromosomes in birds. We isolated four repetitive sequence families of centromeric heterochromatin from three Anseriformes species, viz., domestic duck (*Anas platyrhynchos*, APL), bean goose (*Anser fabalis*, AFA), and whooper swan (*Cygnus cygnus*, CCY), and characterized the sequences by molecular cytogenetic approach. The 190-bp APL-*Hae*III and 101-bp AFA-*Hinf*I-S sequences were localized in almost all chromosomes of *A*. *platyrhynchos* and *A*. *fabalis*, respectively. However, the 192-bp AFA-*Hinf*I-L and 290-bp CCY-*Apa*I sequences were distributed in almost all microchromosomes of *A*. *fabalis* and in approximately 10 microchromosomes of *C*. *cygnus*, respectively. APL-*Hae*III, AFA-*Hinf*I-L, and CCY-*Apa*I showed partial sequence homology with the chicken nuclear-membrane-associated (CNM) repeat families, which were localized primarily to the centromeric regions of microchromosomes in Galliformes, suggesting that ancestral sequences of the CNM repeat families are observed in the common ancestors of Anseriformes and Galliformes. These results collectively provide the possibility that homogenization of centromeric heterochromatin occurred between microchromosomes in Anseriformes and Galliformes; however, homogenization between macrochromosomes and microchromosomes also occurred in some centromeric repetitive sequences.

## Introduction

Highly repetitive DNA sequences are one of the major components of chromosomes, which are generally divided into two categories, viz., interspersed repetitive sequences and site-specific repetitive sequences, based on their genomic organization and chromosomal distribution [1]. Two subtypes of non-long terminal repeat (LTR) retrotransposons, viz., long interspersed elements (LINEs) and short interspersed elements (SINEs), are well-known as major components of interspersed-type repetitive sequences, which are distributed throughout the genome. Site-specific highly repetitive sequences constituting the heterochromatin viz., centromeric repetitive DNA sequences, non-centromeric chromosome site-specific repetitive sequences, microsatellite repeat motifs, etc., are tandem duplicated and are usually present as more than 10,000 copies in the genome, which have important roles in chromosome organization, sex chromosome differentiation, and chromatin architecture in interphase nuclei [2–4]. Centromeric heterochromatin-associated highly repetitive DNA sequences have been isolated and characterized from a high number of vertebrates. These sequences are generally susceptible to rapid nucleotide substitution; therefore, they often rapidly evolve in a concerted manner, resulting in low and intraspecific sequence variation but a higher degree of interspecific sequence variation [5–7]. This indicates that the centromeric repetitive sequence is a good taxonomic and phylogenetic marker for reconstructing the evolutionary relationships between closely related species that share the same origin of repetitive sequence families.

The typical avian karyotype consists of 6–10 pairs of macrochromosomes, including sex chromosomes, and a number of indistinguishable microchromosomes, with diploid numbers ranging from 74 to 84 [8–10]. Genome sequencing of many avian species, including chicken (*Gallus gallus*, Galliformes), has revealed that there are distinct structural differences between macrochromosomes and microchromosomes in terms of multiple factors such as of recombination rate, GC and CpG contents, gene density, and density of repetitive sequences [11–13]. In Galliformes, repetitive sequences distributed primarily on microchromosomes have been isolated from chicken [chicken nuclear-membrane-associated (CNM) repeat] [14], the Japanese quail (*Coturnix japonica*; CJA-*Bgl*II) [15], turkey (*Melleagris gallopavo*; TM repeat) [16], the Blue-breasted Quail (*Coturnix chinensis*; CCH-S) [17], bobwhite quail (*Colinus virginianus*; CVI-*Msp*I) [18], and chukar partridge (*Alectoris chukar*; ACH-*Sau3A*I) [18]. In other avian and reptilian species, microchromosome-specific centromeric repetitive sequences or microsatellite repeat motifs on microchromosomes have also been observed the lesser rhea (*Pteronemia pennata*) and greater rhea (*Rhea americana*), of Struthioniformes [19]; Japanese mountain hawk-eagle (*Nisaetus nipalensis orientalis*), of Falconiformes [20]; campo flicker (*Colaptes campestris*), green-barred woodpecker (*C*. *melanochloros*), and white woodpecker (*melanerpes candidus*) of Piciformes [21]; and Chinese soft-shelled turtle (*Pelodiscus sinensis*) [22]. These results suggest that the centromeric repetitive sequences are homogenized predominantly between microchromosomes in Aves and turtles, known as chromosome size-correlated genomic compartmentalization. Among these microchromosome-specific repeat families, four CNM repeat families (CNM repeat, TM repeat, CCH-S, and ACH-*Sau3A*I) contain 12–17-bp T-rich and A-rich motifs, in which the conserved A_3–5_ and T_3–5_ reiterations are separated by 6–7 bp [14,16,18]. This result indicates that the CNM repeat families originate from the same ancestral repetitive sequence in the common ancestor. However, chromosome size-correlated compartmentalization is collapsed in several avian species, in which the centromeric repetitive sequences localized to both macrochromosomes and microchromosomes have been identified [18,20,23,24]. Therefore, molecular cytogenetic characterization of the centromeric repetitive sequences in other avian orders provides important information on the evolutionary changes in genomic organization of avian macrochromosomes and microchromosomes.

The order Anseriformes consists of three extant families, viz., Anatidae, Anhimidae, and Anseranatidae, containing over 180 species; they are highly adapted for swimming on the surface of water. Anseriformes is considered to have appeared approximately 77 million years ago when the ancestral Galloanserae split into the two main lineages of Anseriformes and Galliformes [25]. Anseriformes and Galliformes are the most basal lineages of neognathous birds that appeared after Palaeognathae in the phylogeny of birds. The karyotypes of Anseriformes are characterized by high diploid chromosome numbers (2n = 78–98), and their typical karyotypes are composed of 78–80 chromosomes that consist of a small number of macrochromosomes and numerous microchromosomes, which are similar to typical avian karyotypes [8–10,26]. The tandem repetitive sequence, RBMII, which was isolated from the Red-breasted Merganser (*Mergus serrator*) [27], was found in all 22 Anseriformes species, including the domestic duck (*A*. *platyrhynchos*) [28]. However, in Anseriformes, molecular characterization of repetitive sequences is limited to RBMII sequences, whose chromosomal distribution is not known yet.

In this study, to improve our understanding of chromosome size-correlated genomic compartmentalization in Aves, we isolated repetitive sequences constituting centromeric heterochromatin from three Anseriformes species, viz., the domestic duck (*A*. *platyrhynchos*), bean goose (*A*. *fabalis*), and whooper swan (*C*. *cygnus*), and characterized the sequences molecular cytogenetically. We examined the chromosomal locations, genomic organization, and sequence conservation among the avian species using fluorescent in situ hybridization (FISH) and filter hybridization. Finally, we discussed the molecular evolution of the centromeric repetitive sequences in Anseriformes and genomic compartmentalization between macrochromosomes and microchromosomes in Aves based on the obtained data.

## Materials and methods

### Ethics statement

Animal care and all experimental procedures were conducted according to the guidelines for the care and use of experimental animals of Nagoya University. The animal protocols were approved by the Animal Experiment Committee, Graduate School of Bioagricultural Sciences, Nagoya University (approved no. 2009090901).

### Cell culture, chromosome preparation, and chromosome banding

For cell culture, small pieces of skin tissues were collected from a 1-month-old female domestic duck (*A*. *platyrhynchos*, Anatidae), purchased from a breeding farm in Japan, and a female bean goose (*A*. *fabalis*, Anatidae) and two female whooper swans (*C*. *cygnus*, Anatidae) from the Asahiyama Zoo, Asahikawa and Kushiro Zoo, Kushiro, respectively, Hokkaido, Japan. Fibroblasts were cultured in Medium 199 (Thermo Fisher Scientific-GIBCO, Carlsbad, CA, USA) supplemented with 15% fetal bovine serum (Thermo Fisher Scientific-GIBCO), 100 μg/ml kanamycin, and 1% antibiotic–antimycotic solution (PSA) (Thermo Fisher Scientific-GIBCO) at 39°C in 5% CO_2_. For Giemsa-stained and C-banded karyotype analyses, fibroblasts were collected 30 min after colcemid treatment, suspended in 0.075 M KCl for 20 min, and then fixed with 3:1 methanol/acetic acid. Chromosome preparations were prepared following the standard air-drying method. Replication-banded chromosome slides were prepared for in situ hybridization as described previously [29]. The cultured cells were treated with 5-bromodeoxyuridine (BrdU) (25 μg/ml) (Sigma-Aldrich, St Louis, MO, USA) at the late replication stage for 4.5 h, including the 30-min colcemid treatment. After staining the slides with Hoechst 33258 (1 μg/ml) for 5 min, replication bands were obtained by heating at 65°C for 3 min and exposing to UV light at 65°C for an additional 6 min. The slides were maintained at −80°C until use.

To examine the chromosomal distribution of centromeric heterochromatin, C-banding was performed by the barium hydroxide/saline/Giemsa method [30] with slight modification; chromosome slides were treated with 0.2 M HCl for 5 min at room temperature and then with 5% Ba(OH)_2_ at 50°C for 5–7 min.

### FISH

For cross-species chromosome painting, we used DNA probes of chicken (*G*. *gallus*, GGA) chromosomes 1–9 and Z [31] and a mixture of microchromosome-specific paints, consisting of 20 pairs of chicken microchromosomes [32] as described previously [33]. FISH probes were labeled with biotin-16-dUTP (Roche Diagnostics, Basel, Switzerland) by nick translation and hybridized to metaphase spreads. After washing, the slides were incubated with Avidin, Alexa Fluor 488 conjugate (Thermo Fisher Scientific-Molecular Probes, Carlsbad, CA, USA) and stained with 0.75 μg/ml propidium iodide (PI). For dual-color FISH, the other probe was labeled with digoxigenin (DIG)-11-dUTP (Roche Diagnostics) and stained with rhodamine-conjugated anti-DIG Fab fragments (Roche Diagnostics) [34].

### Molecular cloning and nucleotide sequencing of repetitive DNA sequences

High molecular weight genomic DNA was extracted from the liver of female *A*. *platyrhynchos* and cultured fibroblasts of female *A*. *fabalis* and *C*. *cygnus*. Genomic DNA of *A*. *platyrhynchos* was digested with 10 restriction endonucleases, viz., *Alu*I, *BamH*I, *Bgl*II, *EcoR*I, *Hae*III, *Hind*II, *Msp*I, *Sae*I, *Sma*I, and *Taq*I. Genomic DNA of *A*. *fabalis* and *C*. *cygnus* was digested with 19 restriction endonucleases, viz., *Apa*I, *BamH*I, *Bgl*I, *Bgl*II, *EcoR*I, *EcoR*V, *Hae*III, *Hind*III, *Hinf*I, *Nsi*I, *Pvu*II, *Rsa*I, *Sac*I, *Sau3A*I, *Sma*I, *Sph*I, *Taq*I, *Xba*I, and *Xho*I. The digested genomic DNA was size-fractionated by 3% agarose gel electrophoresis and then stained with ethidium bromide. Prominent DNA bands of repetitive sequences were eluted from the gel using the QIAquick Gel Extraction Kit (Qiagen, Hilden, Germany) and cloned into the pGEM-7f(+) vector (Promega, Madison, WI, USA). Nucleotide sequences were determined using ABI PRISM 3130 DNA Analyzer (Thermo Fisher Scientific-Applied Biosystems, Carlsbad, CA, USA) after cycle-sequencing reactions with the BigDye Terminator v3.1 Cycle Sequencing Kit (Thermo Fisher Scientific-Applied Biosystems). Dot matrix analysis of the nucleotide sequences was performed with MAFFT version 7 (http://mafft.cbrc.jp/alignment/server/).

### Southern blot hybridization

Genomic DNA was digested with restriction endonucleases, fractionated on 2% agarose gel, and transferred onto nylon membranes (Roche Diagnostics). DNA fragments of repetitive sequences were labeled with DIG-11-dUTP using PCR DIG Labeling Mix (Roche Diagnostics) and hybridized to the membrane. Hybridization was performed overnight at 45°C in DIG Easy Hyb Solution (Roche Diagnostics). After hybridization, the membrane was washed at 45°C in 0.1% sodium dodecyl sulfate (SDS)/2× saline sodium citrate (SSC), 0.1% SDS/1× SSC, 0.1% SDS/0.5× SSC, and 0.1% SDS/0.1× SSC for 15 min each. Chemiluminescent signals were detected with anti-DIG-AP Fab fragments and CDP-Star (Roche Diagnostics) and exposed to Biomax MS-1 Autoradiography Film (Carestream Health, Rochester, NY, USA).

### Slot blot hybridization

To examine the nucleotide sequence conservation of repetitive sequences among avian species, slot blot hybridization was performed with DIG-11-dUTP-labeled DNA fragments as described in our previous studies [18,35]. We used genomic DNA from females of 17 avian species representing 10 orders, viz., ostrich (*Struthio camelus*) and emu (*Dromaius novaehollandiae*) belonging to Struthioformes; elegant crested tinamou (*Eudromia elegans*) belonging to Tinamiformes; helmeted guineafowl (*Numida meleagris*), Japanese quail (*C*. *japonica*), and chicken (*G*. *gallus*) belonging to Galliformes; bean goose (*A*. *fabalis*), whooper swan (*C*. *cygnus*), and domestic duck (*A*. *platyrhynchos*) belonging to Anseriformes; Siberian crane (*Grus leucogeranus*) belonging to Gruiformes; Black-faced Spoonbill (*Platalea minor*) belonging to Pelecaniformes; Blakiston’s fish owl (*Bubo blakistoni*) belonging to Strigiformes; osprey (*Pandion haliaetus*) and Japanese mountain hawk-eagle (*N*. *nipalensis orientalis*) belonging to Accipitriformes; palm cockatoo (*Probosciger aterrimus*) and Yellow-naped Amazon (*Amazona auropalliata*) belonging to Psittaciformes; and barn swallow (*Hirundo rustica*) belonging to Passeriformes. RNase-treated whole genomic DNA (500 ng) was denatured with 0.4 N NaOH for 10 min and transferred to nylon membranes (Roche Diagnostics) using BIO-DOT SF blotting equipment (Bio-Rad, Hercules, CA, USA). DNA probes of repetitive sequences were labeled with DIG-11-dUTP using the PCR DIG Labeling Mix (Roche Diagnostics) and hybridized with the membranes at 45°C in DIG Easy Hyb solution (Roche Diagnostics). Chemiluminescent signals were detected as described in Southern blot hybridization.

## Results

### Giemsa-stained and C-banded karyotypes

**Fig 1:**
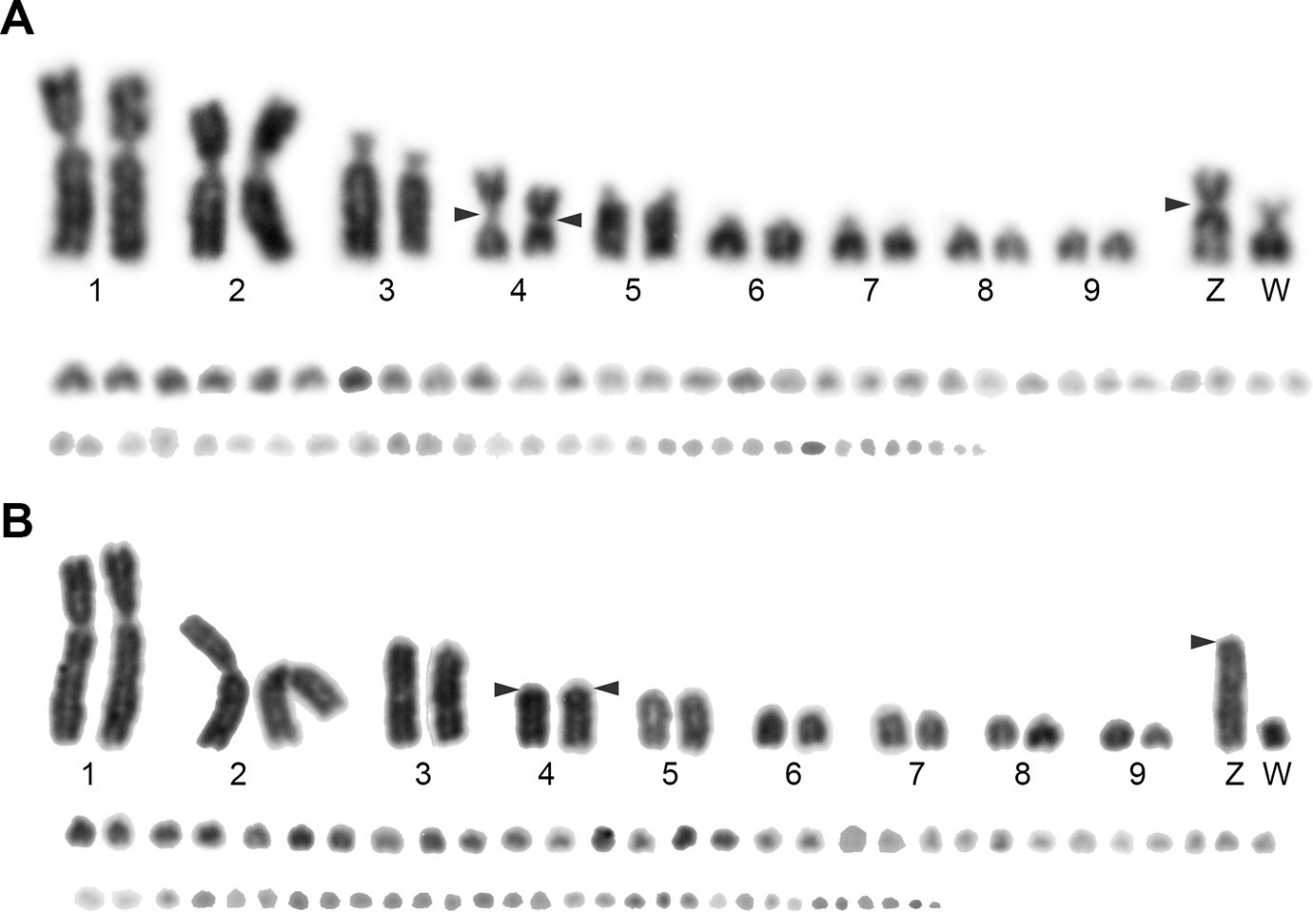
**Giemsa-stained karyotypes of female bean goose (*A*. *fabalis*) (A) and whooper swan (*C*. *cygnus*) (B)**. Arrowheads indicate the positions of the centromeres in chromosome 4 and the Z and W sex chromosomes.

**Fig 2:**
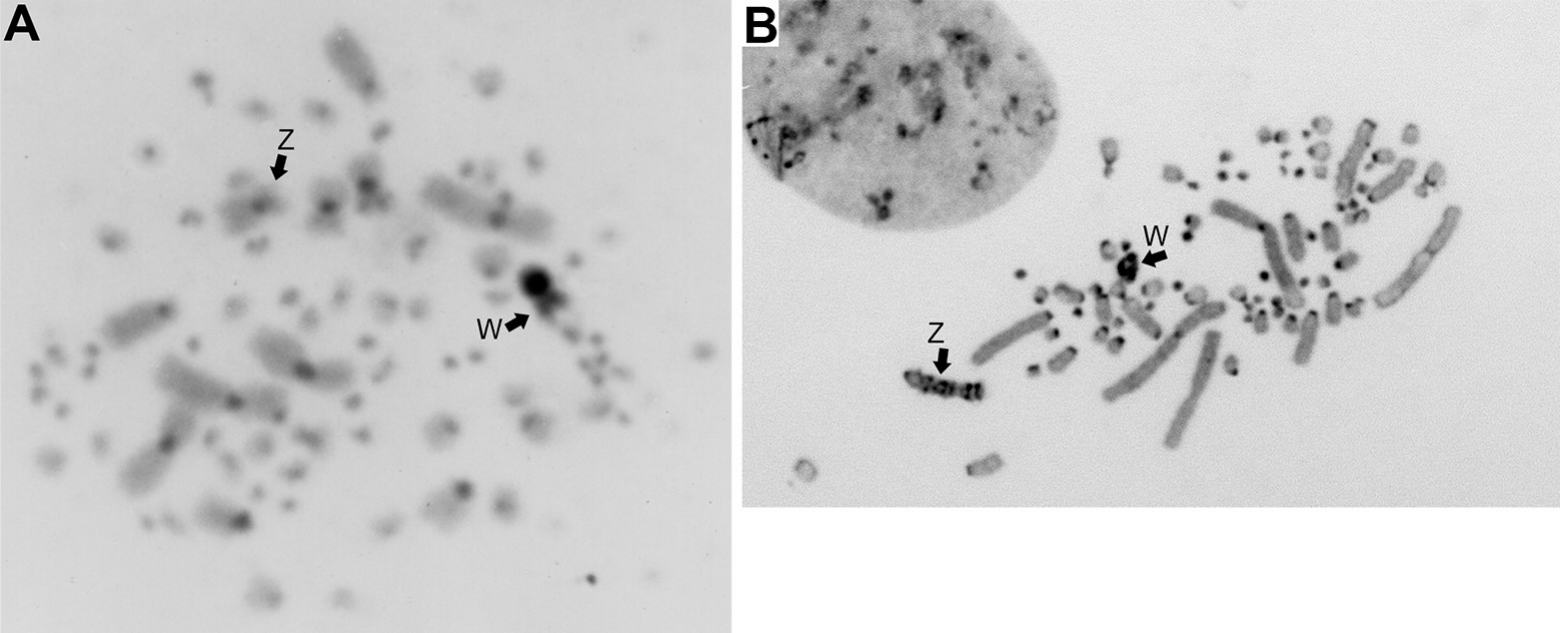
**C-banded metaphase spreads of *A*. *fabalis* (A) and *C*. *cygnus* (B) females.** Arrows indicate the Z and W sex chromosomes.

Giemsa-stained and C-banded karyotypes of *A*. *platyrhynchos* (2n = 80) were described in previous studies [36–41]. The chromosome number of *A*. *fabalis* was 2n = 80, consisting of two pairs of large submetacentric chromosomes, one pair each of large subtelocentric chromosomes, medium-sized metacentric chromosomes, and medium-sized subtelocentric chromosomes; four pairs of small acrocentric chromosomes; 30 pairs of indistinguishable microchromosomes; and the submetacentric Z and subtelocentric W sex chromosomes (Fig 1A). The karyotype of *C*. *cygnus* (2n = 80) consisted of one pair each of large submetacentric chromosomes, large metacentric chromosomes, and large acrocentric chromosomes; two pairs of medium-sized acrocentric chromosomes; four pairs of small acrocentric chromosomes; 30 pairs of indistinguishable microchromosomes; and the acrocentric Z and small acrocentric W sex chromosomes, which were similar to those of *A*. *platyrhynchos* (Fig 1B). The morphology of the chromosomes 4, Z, and W chromosomes was acrocentric in *A*. *platyrhynchos* and *C*. *cygnus*, whereas these three chromosomes were metacentric, submetacentric, and subtelocentric, respectively, in *A*. *fabalis* (Fig 1). Large C-positive heterochromatin blocks were observed in the centromeric regions of most autosomes and the Z chromosome and in whole regions of the W chromosome in these two species as well as *A*. *platyrhynchos* (Fig 2) [38,39,41]. In *A*. *platyrhynchos* and *A*. *fabalis*, C-positive heterochromatin on the Z chromosome was observed in only the centromeric region (Fig 2A), whereas ladder C-positive heterochromatin blocks were observed throughout the region of the Z chromosome in *C*. *cygnus* (Fig 2B).

### Chromosome homologies with chicken chromosomes

**Fig 3:**
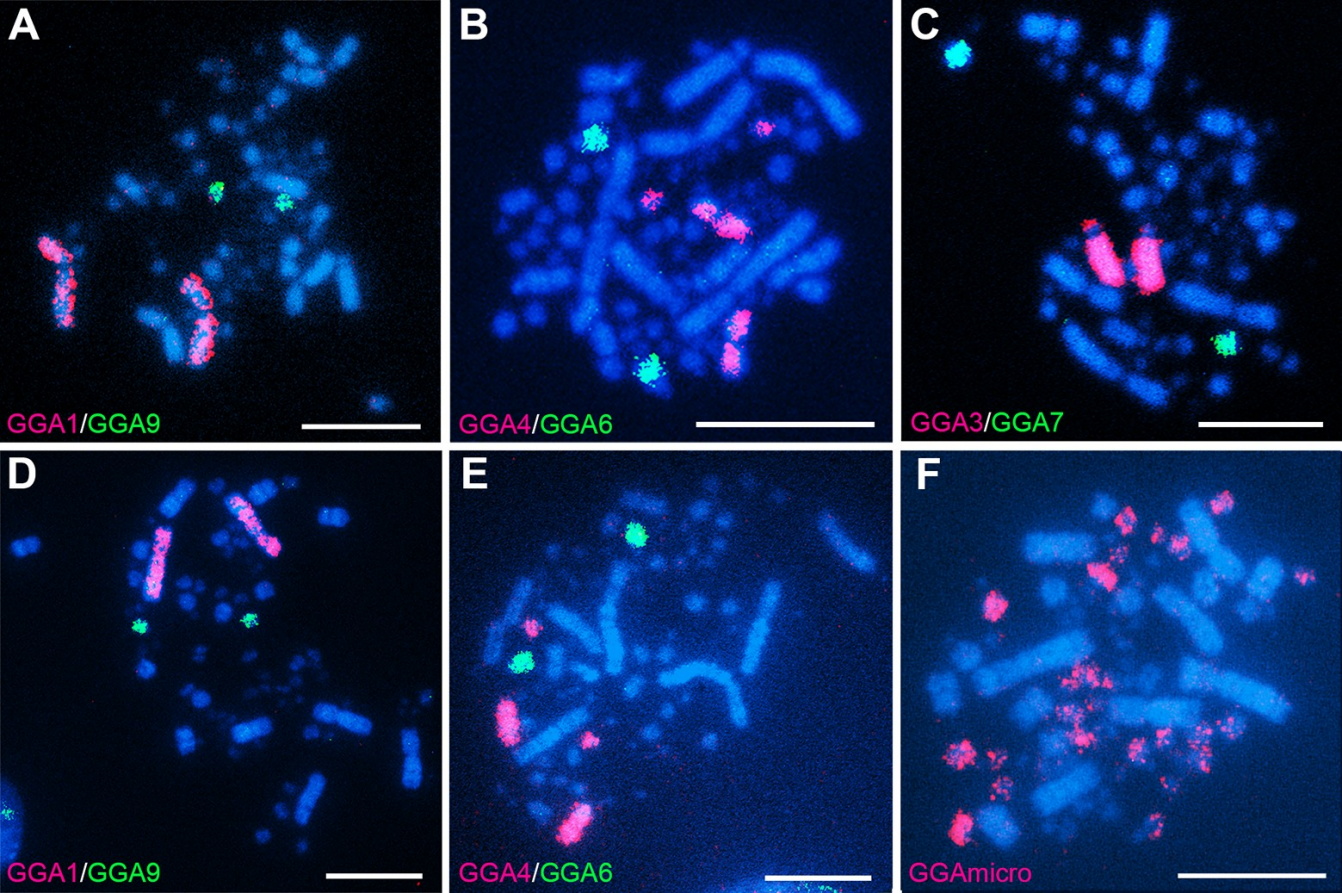
**Chromosome painting with chicken (*G*. *gallus*, GGA) chromosome-specific DNA probes to Hoechst-stained chromosome spreads of *A*. *fabalis* (A‒C) and *C*. *cygnus* females (D‒E).** DIG-labeled GGA1 (red) and biotin-labeled GGA9 (green) hybridized to chromosomes 1 and 9, respectively (A, D). DIG-labeled GGA4 (red) hybridized to chromosome 4 and a pair of microchromosomes, and biotin-labeled GGA6 (green) hybridized to chromosome 6 (B, E). DIG-labeled GGA3 (red) hybridized to chromosome 3, and biotin-labeled GGA7 (green) hybridized to chromosome 7 (C). DIG-labeled painting probe of 20 microchromosome pairs hybridized to approximately half of the microchromosomes (F). Scale bars represent 10 μm.

Each of macrochromosome probes (GGA1–3, 5–9, and Z), except for GGA4, painted a single pair of chromosomes of *A*. *fabalis* and *C*. *cygnus* (Fig 3). The GGA4 probe hybridized to chromosome 4 and additionally to one pair of microchromosomes (Fig 3B and 3E). These results of *A*. *fabalis* and *C*. *cygnus* were consistent with those of *A*. *platyrhynchos* in our previous study [40]. The microchromosome-specific paint pool (GGAmicro) hybridized with approximately half of microchromosomes, and no hybridization signals were detected on macrochromosomes (Fig 3F).

### Repetitive sequence families and their nucleotide sequences

Prominent DNA bands of repetitive sequences were revealed by agarose gel electrophoresis of *A*. *platyrhynchos* (APL) genomic DNA digested with *Hae*III. A DNA band of approximately 190 bp was isolated from the gel, and then 16 clones inserted into plasmid vectors were obtained. For *A*. *fabalis* (AFA), 16 and 8 clones were obtained from two bands of approximately 100 and 200 bp by *Hinf*I digestion, respectively. For *C*. *cygnus*, 50 clones were obtained from a band of approximately 300 bp by *Apa*I digestion. Nucleotide sequences were determined for all clones.

Consequently, 26 DNA fragments categorized into four families of repetitive sequences were isolated from these three species and deposited in DDBJ (http://www.ddbj.nig.ac.jp/), viz., the APL-*Hae*III family from *A*. *platyrhynchos*, the AFA-*Hinf*I-S and AFA-*Hinf*I-L familiy from *A*. *fabalis*, and the CCY-*Apa*I family from *C*. *cygnus* (Table 1). The sizes, GC content, and nucleotide sequence identities between the fragments and within the same sequence family are summarized in Table 1. The length of the consensus sequence of five APL-*Hae*III fragments was 190 bp (S1 Fig). A deletion of 29 nucleotides was found at the end of two 161-bp fragments (APL-*Hae*III-08 and APL-*Hae*III-11). Nucleotide sequence identities, which were calculated by eliminating insertions and deletions but including one nucleotide gap, ranged from 78.2% to 89.4% (84.7% on an average), and the GC content was relatively high (51.6% on an average). All four AFA-*Hinf*I-S fragments were of the same length of 101 bp, and two AFA-*Hinf*I-L fragments were of 192 bp (S2 Fig). The identities of nucleotide sequences between the fragments ranged from 91.0% to 98.1% (94.1% on an average) for the AFA-*Hinf*I-S sequence family and 98.4% for the AFA-*Hinf*I-L sequence family. AFA-*Hinf*I-S showed much higher GC content (60.2%) compared to that of AFA-*Hinf*I-L (51.6%). The length of 15 CCY-*Apa*I fragments ranged from 286 to 294 bp with 97.6% sequence identify and 54.0% GC content on an average (S3 Fig).

**Table 1:** Repetitive sequence families isolated from *A*. *platyrhynchos*, *A*. *fabalis*, and *C*. *cygnus*, and their lengths, sequence identities between fragments, and GC content.

Repetitive sequence family	No. of clones	Length of concensus sequence (bp)^a^	Average sequence identity between fragments (%)^b^	Average GC content (%)^c^	Accession number
*A*. *platyrhynchos*					
APL-*Hae*III	5/16	190 (161 ‒ 190)	84.7 (78.2 ‒ 89.4)	51.6 (48.7 ‒ 53.2)	LC416791 –LC416795
*A*. *fabalis*					
AFA-*Hinf*I-S	4/16	101 (101)	94.1 (91.0 ‒ 98.1)	60.2 (58.4 ‒ 61.4)	LC416770 –LC416773
AFA-*Hinf*I-L	2/8	192 (192)	98.4 (98.4)	51.6 (51.0 ‒ 52.1)	LC416774, LC416775
*C*. *cygnus*					
CCY-*Apa*I	15/50	290 (286 ‒ 294)	97.6 (94.9 ‒ 100.0)	54.0 (53.4 ‒ 54.6)	LC416776 –LC416790

^a^Range of fragment lengths in parentheses

^b^Range of sequence identities in parentheses

^c^Range of GC content in parentheses

**Fig 4:**
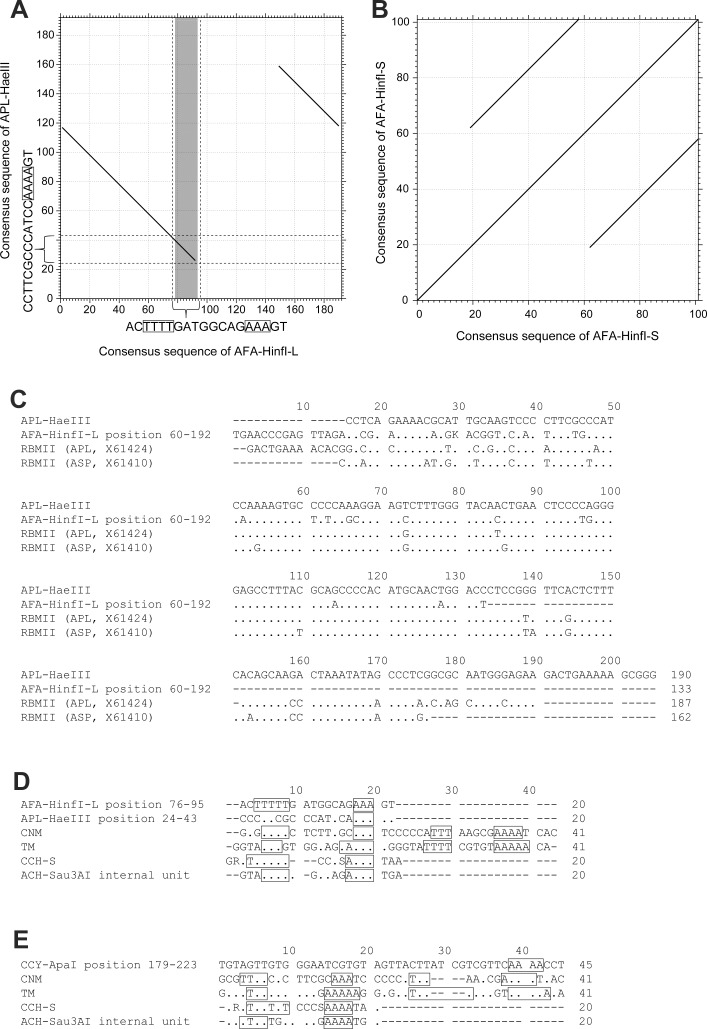
Comparison of the nucleotide sequences of APL-*Hae*III, AFA-*Hinf*I-S, AFA-*Hinf*I-L, and CCY-*Apa*I sequences with their homologous sequences. Dot matrix analysis between the consensus sequences of 190-bp APL-*Hae*III and 192-bp AFA-*Hinf*I-L (A). The gray region on AFA-*Hinf*I-L shows the 12–17-bp T-rich and A-rich motif that is conserved in the CNM repeat sequence family of Galliformes [14,16,18], and squares on the sequences indicate the A_3–5_ or T_3–5_ internal repeats in this motif. Dot matrix analysis of the 101-bp AFA-*Hinf*I-S consensus sequence (B). Dot matrix analysis was performed in the condition of the scoring matrix, 200PAM/K = 2 and threshold score = 22 (E = 0.00805). Alignment of the APL-*Hae*III consensus sequence and partial sequence at nucleotide position 60–192 of the AFA-*Hinf*I-L consensus sequence with the RBMII sequences of *A*. *platyrhynchos* (APL) (X61424) and *Aix sponsa* (ASP) (X61410) (C). Alignment of the partial sequences at nucleotide position 76–95 of the AFA-*Hinf*I-L consensus sequence and at position 24–43 of the APL-*Hae*III consensus sequence (D) and the partial sequence at positions 179–223 of the CCY-*Apa*I consensus sequence (E) with the four CNM sequence homologs in Galliformes, viz., CNM repeat in chicken [14], TM repeat in turkey (*M*. *gallopavo*) [16], CCH-S in Blue-breasted Quail (*C*. *chinensis*) [17], and ACH-*Sau3A*I in chukar partridge (*A*. *chukar*) [18]. Squares indicate the A_3–5_ or T_3–5_ internal repeats in the 12–17-bp T-rich and A-rich motifs conserved in the CNM repeat sequence family of Galliformes [14,16,18].

The nucleotide sequence of AFA-*Hinf*I-L showed homology with APL-*Hae*III. Two partial sequences at positions 26–117 and 118–159 in the consensus APL-*Hae*III sequence showed 84.9% and 76.5% identities with those at positions 1–92 and 149–190 in the consensus AFA-*Hinf*I-L, respectively (Fig 4A). The AFA-*Hinf*I-S sequence contained two 42–43-bp internal repeat units (Fig 4B). Homology searches of the four repetitive sequence families were performed using the NCBI non-redundant sequence database (http://blast.ncbi.nlm.nih.gov) and Repbase (http://www.girinst.org/repbase/). The consensus sequences of APL-*Hae*III and the partial sequence of AFA-*Hinf*I-L at positions 60–192 showed similarity with the RBMII sequences isolated from several Anseriformes species, such as *A*. *platyrhynchos* (X61424) (88.0% and 81.4% identity, respectively) and wood duck (*Aix sponsa*, ASP) (X61410) (88.1% and 79.4% identity, respectively) (Fig 4C and S1 Table), indicating that APL-*Hae*III and AFA-*Hinf*I-L shared the same origin as that the of RBMII sequence, which is a major tandem repetitive sequence in the Anseriformes species [27,28]. Nucleotide sequence homology was observed for 20-bp partial sequences at positions 24–43 in APL-*Hae*III and at positions 76–95 in AFA-*Hinf*I-L (26.0%–47.6% and 38.0%–59.0%, respectively) and with the CNM repeat family sequences in Galliformes, chicken CNM repeat [14], turkey TM repeat [16], Blue-breasted Quail CCH-S [17], and chukar partridge ACH-*Sau3A*I (AB872160) [18] (Fig 4D). The highest identity (59.0%) was found between the partial sequence of AFA-*Hinf*I-L and the 20-bp consensus sequence of internal repeat unit of ACH-*Sau3A*I. The T-rich and A-rich motifs of the CNM repeat family were conserved in AFA-*Hinf*I-L but not found in APL-*Hae*III (Fig 4A and 4D). The partial sequence at position 179–223 of CCY-*Apa*I also showed homology with the CNM repeat family sequences of Galliformes (35.0%–55.0%; Fig 4E). The highest identity (55.0%) was observed for the TM repeat of turkey [14]; however, the T-rich and A-rich motifs were not detected in the 45-bp partial sequence of CCY-*Apa*I.

### Chromosomal distribution

**Fig 5:**
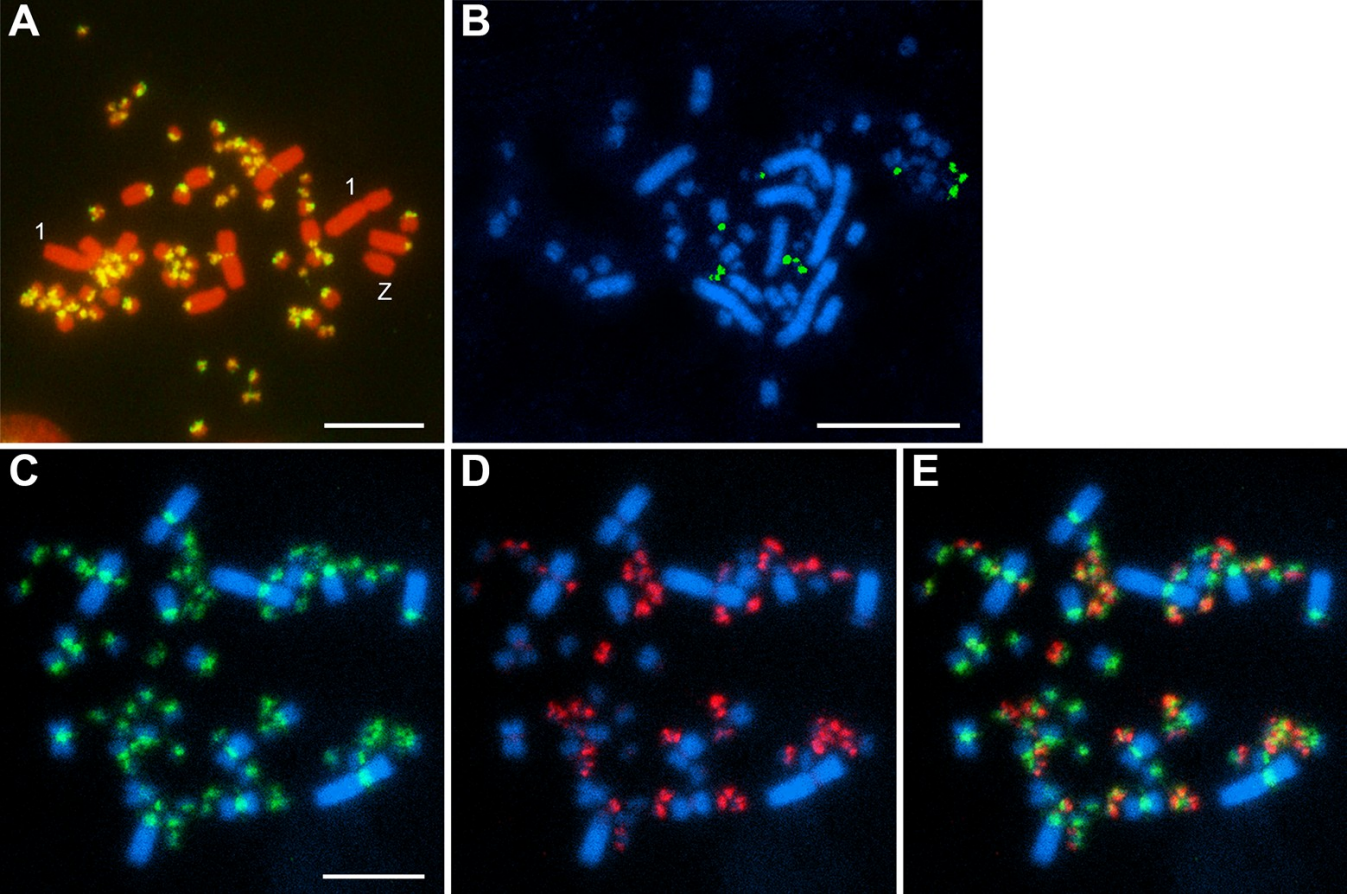
Chromosomal distribution of four families of repetitive sequences on metaphase spreads. Chromosomal distribution of the biotin-labeled APL-*Hae*III-04 fragment on the PI-stained metaphase chromosome spread of *A*. *platyrhynchos* female (A). Hybridization pattern of the biotin-labeled CCY-*Apa*I-05 fragment to the Hoechst-stained metaphase spread of *C*. *cygnus* female (B). Hybridization patterns of the biotin-labeled AFA-HinfI-S03 fragment (green) (C) and DIG-labeled AFA-*Hinf*I-L04 fragment (red) (D) to the Hoechst-stained metaphase spread of *A*. *fabalis* female and their merged image (E). Scale bars represent 10 μm.

In *A*. *platyrhynchos*, the APL-*Hae*III sequence family showed intense hybridization signals in the centromeric regions of all macrochromosomes and microchromosomes, except for the chromosomes 1 and Z, i.e., no signals were observed on the chromosomes 1 and Z (Fig 5A). In *C*. *cygnus*, CCY-*Apa*I was localized to the centromeric regions of approximately 10 microchromosomes (Fig 5B). AFA-*Hinf*I-S and AFA-*Hinf*I-L were localized to the centromeric/pericentromeric regions of all chromosomes and almost all microchromosomes, respectively, in *A*. *fabalis* (Fig 5C–5E). The fluorescent signals of AFA-*Hinf*I-L and AFA-*Hinf*I-S hardly overlapped, suggesting that the two sequence families were located separately in centromeric heterochromatin on the same microchromosomes (Fig 5E).

### Organization in the genome

**Fig 6:**
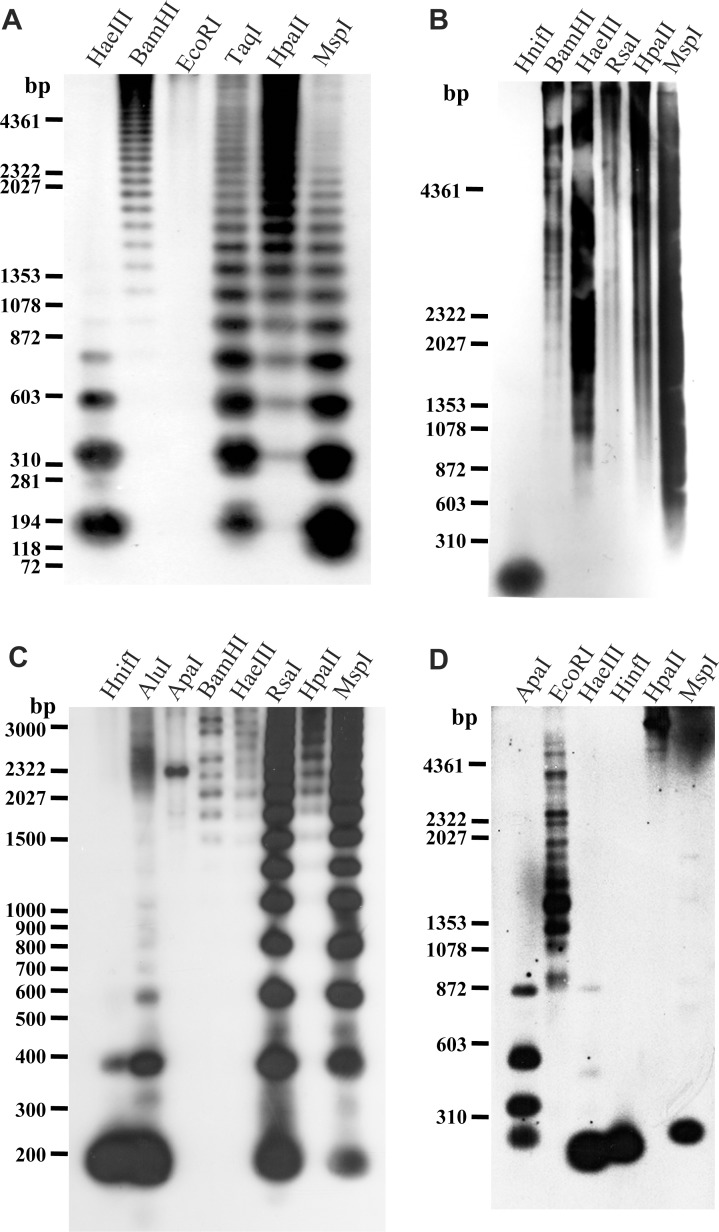
Southern blot hybridization patterns of four repetitive sequence families. Southern blot hybridization of *A*. *platyrhynchos* genomic DNA probed with the APL-*Hae*III-04 fragment (A). Southern blot hybridization of *A*. *fabalis* genomic DNA probed with the AFA-*Hinf*I-S03 (B) and AFA-*Hinf*I-L04 (C) fragments. Southern blot hybridization of *C*. *cygnus* genomic DNA probed with the CCY-*Apa*I-05 fragment (D). A mixture of λ DNA–*Hind*III and ϕX174 DNA–*Hae*III was used as a molecular size marker for (A, B, D), and a mixture of λ DNA–*Hind*III digest and 100-bp ladder digest was used for (C).

Southern blot hybridization was performed to examine the genomic organization of four families of repetitive sequences. APL-*Hae*III showed polymeric ladder signals of tandem repeats of the 190-bp monomer unit in *Hae*III, *Msp*I, and *Taq*I digests (Fig 6A); the monomer unit was present in the highest abundance, with decreasing copy numbers of each higher order. By contrast, the *BamH*I digest produced higher intensity of hybridization bands with increasing size of multimers. This result indicated that the *BamH*I cleavage site was not highly conserved in the tandem array of the 190-bp monomer unit. The restriction site for both *Hpa*II and *Msp*I is CCGG, and *Hpa*II does not cleave when the second cytosine is methylated, whereas *Msp*I does. In contrast to the *Msp*I digest, the intensity of ladder bands increased from low to high molecular weight in *Hpa*II digests, indicating that the APL-*Hae*III sequence was highly methylated.

Hybridization of the AFA-*Hinf*I-S sequence showed only a 101-bp monomeric band in the *Hinf*I digest, indicating that the *Hinf*I site was highly conserved with regard to the tandem array of the sequence (Fig 6B). However, no restriction sites of *BamH*I, *Hae*III, *Rsa*I, and *Msp*I were found in the AFA-*Hinf*I-S monomer unit (S2A Fig), thus, resulting in smear-like bands in the digests of these enzymes. This sequence was weakly methylated because the intensities of ladder bands at lower molecular weight were slightly higher in the *Hpa*II digest than those in the *Msp*I digest.

In hybridization with AFA-*Hinf*I-L, ladder bands at lower molecular weight were observed in the *Hinf*I, *Alu*I, *Rsa*I, and *Msp*I digests, whose restriction sites were all contained in the 192-bp monomer unit (Fig 6C and S2 Fig). Of these sites, the *Hinf*I and *Alu*I sites were particularly highly conserved. In contrast to the *Msp*I digest, there were no hybridization signals at a lower molecule weight in the *Hpa*II digest, indicating that this sequence was hypermethylated.

Hybridization of the 290-bp CCY-*Apa*I fragment revealed that the *Apa*I, *Hinf*I, and *Msp*I sites were conserved in the tandem array of this repeat sequence (Fig 6D and S3 Fig). The hybridization bands positioned at approximately 580 and 870 bp corresponded to the dimeric and trimeric bands in the *Apa*I digest, respectively. The intermediate band between the 290- and 580-bp bands may have been derived from the internal restriction sites; however, this site was not found for this fragment. The <290-bp hybridization band in the *Hae*III digest was derived from multiple internal *Hae*III sites contained in the sequence (S3 Fig). Only one 290-bp band was observed in the *Msp*I digest; however, no bands were found at lower molecular weight in the *Hpa*II digest, indicating that this repetitive sequence was hypermethylated.

### Nucleotide sequence conservation

**Fig 7:**
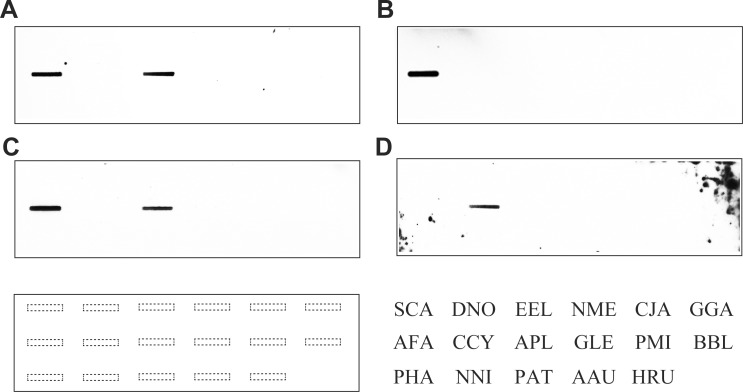
Slot blot hybridization of four repetitive sequences to genomic DNA of 17 species. The fragments used for this experiment are as follows: APL-*Hae*III-04 (A), AFA-*Hinf*I-S03 (B), AFA-*Hinf*I-L04 (C), and CCY-*Apa*I-05 (D). Genomic DNA used for this experiment was obtained from the following avian species of 10 orders: (1) Struthioniformes, SCA (*S*. *camelus*) and DNO (*D*. *novaehollandiae*); (2) Tinamiformes, EEL (*E*. *elegans*); (3) Galliformes, NME (*N*. *meleagris*), CJA (*C*. *japonica*), and GGA (*G*. *gallus*); (4) Anseriformes, AFA (*A*. *fabalis*), CCY (*C*. *cygnus*), and APL (*A*. *platyrhynchos*); (5) Gruiformes, GLE (*G*. *leucogeranus*); (6) Pelecaniformes, PMI (*P*. *minor*); (7) Strigiformes, BBL (*B*. *blakistoni*); (8) Accipitriformes, PHA (*P*. *haliaetus*) and NNI (*N*. *nipalensis orientalis*); (9) Psittaciformes, PAT (*P*. *aterrimus*) and AAU (*A*. *autumnalis*); and (10) Passeriformes, HRU (*H*. *rustica*). The locations of genomic DNA on membranes used for slot blot hybridization are represented at the bottom of the figures.

Slot blot hybridization probed with four families of repetitive sequences was performed using genomic DNA from 17 avian species of 10 orders (Fig 7). Hybridization signals of APL-*Hae*III and AFA-*Hinf*I-L were detected for two species of Anseriformes, viz., *A*. *platyrhynchos* and *A*. *fabalis* (Fig 7A and 7C). The hybridization signals of AFA-*Hinf*I-S and CCY-*Apa*I were detected only in *A*. *fabalis* and *C*. *cygnus*, respectively (Fig 7B and 7D). In order to examine the chromosomal distribution of AFA-*Hinf*I-L in *A*. *platyrhynchos* and APL-*Hae*III in *A*. *fabalis*, we performed cross-species FISH mapping of AFA-*Hinf*I-L to *A*. *platyrhynchos* chromosomes and of APL-*Hae*III to *A*. *fabalis* chromosomes. However, no hybridization signals were found.

## Discussion

Giemsa-stained karyotype analysis revealed that the chromosome number was 2n = 80 for both *A*. *fabalis* and *C*. *cygnus* as *A*. *platyrhynchos* [36–41]. The size and morphology of most macrochromosomes were similar between these two species; however, the chromosomes 4, Z, and W of *A*. *platyrhynchos* and *C*. *cygnus* were morphologically different from those of *A*. *fabalis*. C-positive heterochromatin was observed in the centromeric regions of almost all autosomes and whole regions of the W chromosomes in *A*. *fabalis* and *C*. *cygnus*, which was consistent with that of *A*. *platyrhynchos*, as reported previously [39,41]. However, amplification of the interstitial heterochromatin blocks in the Z chromosome, as shown by the C-positive ladder signals, occurred specifically in the lineage of *C*. *cygnus*.

The chicken chromosome paints 1–9 and Z each hybridized to a single pair of chromosomes in *A*. *fabalis* and *C*. *cygnus*, except for GGA4 that hybridized to a single pair of macrochromosomes (chromosome 4) and a single pair of microchromosomes. The same result has been reported in four other Anseriformes species [swan goose (*Anser cygnoides*), Muscovy duck (*Cairina moschata*), *A*. *platyrhynchos*, and coscoroba swan (*Coscoroba coscoroba*)]; several palaeognathous birds, including ostrich and tinamous; and several Galliformes species, including pheasants, turkey, and New World quail [18,26,33,42–46], indicating that all macrochromosomal structures are highly conserved in Anseriformes, Galliformes, and Palaeognathae. However, GGA4 paint hybridized to a single pair of macrochromosomes (chromosome 4) in one Anseriformes species, greylag goose (*A*. *anser*) [43], and chicken and several phasianid species, viz., chukar partridge (*A*. *chukar*), Japanese quail (*C*. *japonica*), Blue-breasted Quail (*C*. *chinensis*), Chinese bamboo-partridge (*Bambusicola thoracica*), and common peafowl (*Pavo cristatus*) [18,4,45], indicating that the fusion of a microchromosome to the ancestral chromosome 4 occurred independently in each lineage of Galliformes and Anseriformes after they split from the common ancestor of Galloanserae.

In this study, we isolated four families of centromere-specific repetitive sequences, APL-*Hae*III, AFA-*Hinf*-S, AFA-*Hinf*-L, and CCY-*Apa*I, from three Anseriformes species. Among these four repetitive sequences, AFA-*Hinf*-S and CCY-*Apa*I were species-specific, suggesting that these repetitive sequences occurred independently in each species. APL-*Hae*III and AFA-*Hinf*-L were conserved in *A*. *platyrhynchos* and *A*. *fabalis*, both of which showed sequence similarities to the RBMII sequence that is conserved in at least 22 Anseriformes species [27,28]. However, these two sequence families showed different chromosomal distribution, and no hybridization signals were detected on chromosomes by cross-species FISH mapping. These results indicate that APL-*Hae*III and AFA-*Hinf*-L were derived from the same origin as the RBMII sequence in Anseriformes; however, they differentiated to the extent that they hybridized interspecifically by slot blot hybridization but not by FISH.

APL-*Hae*III and AFA-*Hinf*I-S were distributed in almost all chromosomes. However, AFA-*Hinf*I-L and CCY-*Apa*I were microchromosome-specific centromeric repeats that were firstly identified in Anseriformes. AFA-*Hinf*I-L was predominantly localized to all microchromosomes and CCY-*Apa*I to some microchromosomes. All the four repetitive sequences were Anseriformes-specific sequences; however, the partial sequences of APL-*Hae*III, AFA-*Hinf*I-L, and CCY-*Apa*I showed homology with the CNM family sequences, including chicken CNM, turkey TM, Blue-breasted quail CCH-S, and chukar partridge ACH-*Sau3A*I sequences, which are localized primarily to microchromosomes in Galliformes [14,16–18]. The T-rich and A-rich motifs conserved in the CNM family sequences were observed in AFA-*Hinf*I-L but not in APL-*Hae*III and CCY-*Apa*I. Consequently, the CNM family sequences of Galliformes and APL-*Hae*III, AFA-*Hinf*I-L, and CCY-*Apa*I were concluded to be partially derived from the same ancestral sequence and diverged independently in each lineage. Microchromosome-specific repetitive sequences have been isolated from Falconiformes, Galliformes, Piciformes, and Struthioniformes in Aves and from the Chinese soft-shell turtle [14–22], suggesting that chromosome size-correlated genome compartmentalization between macrochromosomes and microchromosomes is common in birds and turtle. AFA-*Hinf*I-L and CCY-*Apa*I of Anseriformes were also homogenized in a chromosome size-correlated manner. However, the homogenization between macrochromosomes and microchromosomes also occurred in APL-*Hae*III and AFA-*Hinf*I-S, as observed in ACH-*Sau3A*I, CVI-*Hae*III, CCA-*BamH*I, and CSQ-*BamH*I in Galliformes [18].

Not much is known about how chromosome size-dependent distribution of the centromeric repetitive sequences evolved in avian genomes. One possible explanation is that such biased distribution of the centromeric repetitive sequences is caused by chromosome positioning in the nuclei. In the interphase nuclei of chicken, turkey, and Japanese quail, microchromosomes are located predominantly in the nuclear interior, and macrochromosomes are primarily located in peripheral parts of the nuclei [32,47–50]. Owing to the spatially different disposition, physical interaction of chromatin between macrochromosomes and microchromosomes may be restricted, resulting in the restriction of homogenization of the centromeric repetitive sequences between different sized chromosomes. However, the molecular basis that is responsible for the spatial structure of the centromeres of macrochromosomes and microchromosomes in the interphase nuclei are not fully understood.

The presence of microchromosomes is a common feature of Aves, Reptilia (sauropsids), except for Crocodilia [51], and also in some amphibian species [52]. Comparative genome and chromosome analyses for amphibians, reptiles, birds, and mammals suggest that ancestral tetrapods and amniotes may have retained many microchromosomes whose linkages are highly conserved in chicken microchromosomes [53–58]. Comparison of the GC content in exonic third codon positions (GC_3_) of genes between macrochromosomes and microchromosomes in several reptilian species, Chinese soft-shell turtle, Japanese four-striped rat snake (*Elaphe quadrivirgata*), central bearded dragon (*Pogona vitticeps*), and green anole (*Anolis carolinensis*) demonstrated that the genes on microchromosomes tend to have higher GC_3_ than those on macrochromosomes, as shown in chicken, suggesting that chromosome size-dependent GC heterogeneity was acquired in the common ancestors of sauropsids [59–62]. Further identification of the microchromosome-specific centromeric repetitive sequences from avian and reptilian species may help clarify the relationship between the genomic organization of microchromosomes and chromosome size-correlated compartmentalization between macrochromosomes and microchromosomes in tetrapods.

## Supporting information

S1 Fig Nucleotide sequences of five APL-*Hae*III fragments isolated from the *Hae*III-digested genomic DNA of *A. platyrhynchos*. Internal restriction sites of endonucleases are represented by the following underlining: *Hae*III, dots and dashes; and *Msp*I, wave. Dots indicate the same nucleotides as those of the consensus sequence shown at the top, and hyphens indicate gaps.
(TIF)

S2 Fig **Alignments of nucleotide sequences of four AFA-*Hinf*I-S fragments (A) and two AFA-*Hinf*I-L fragments (B) isolated from the *Hinf*I-digested genomic DNA of *A*. *fabalis*.** Internal restriction sites of endonucleases are represented by the following underlining: *Alu*I, conventional; *Hinf*I, double; *Msp*I, wave; and *Rsa*I, dots. Dots indicate the same nucleotides as those of the consensus sequence shown at the top, and hyphens indicate gaps.
(TIF)

S3 Fig Alignment of nucleotide sequences of 15 CCY-*Apa*I fragments isolated from the *Apa*I-digested genomic DNA of *C. cygnus*. Internal restriction sites of endonucleases are represented by the following underlining: *Apa*I, bold; *Hae*III, dots and dashes; *Hinf*I, double; and *Msp*I, wave. Dots indicate the same nucleotides as those of the consensus sequence shown at the top, and hyphens indicate gaps.
(TIF)

S1 Table Nucleotide sequences exhibiting homologies with three repetitive sequence families, APL-*Hae*III, AFA-*Hinf*I-L, and AFA-*Hinf*I-S, except for the CNM family sequence. (XLSX)
