# Supplementary figures and images for: Molecular cytogenetic characterization of repetitive sequences comprising centromeric heterochromatin in three Anseriformes species
Source: PLoS One. 2019 Mar 26;14(3):e0214028. doi: 10.1371/journal.pone.0214028 (PMC6435179; doi:10.1371/journal.pone.0214028)

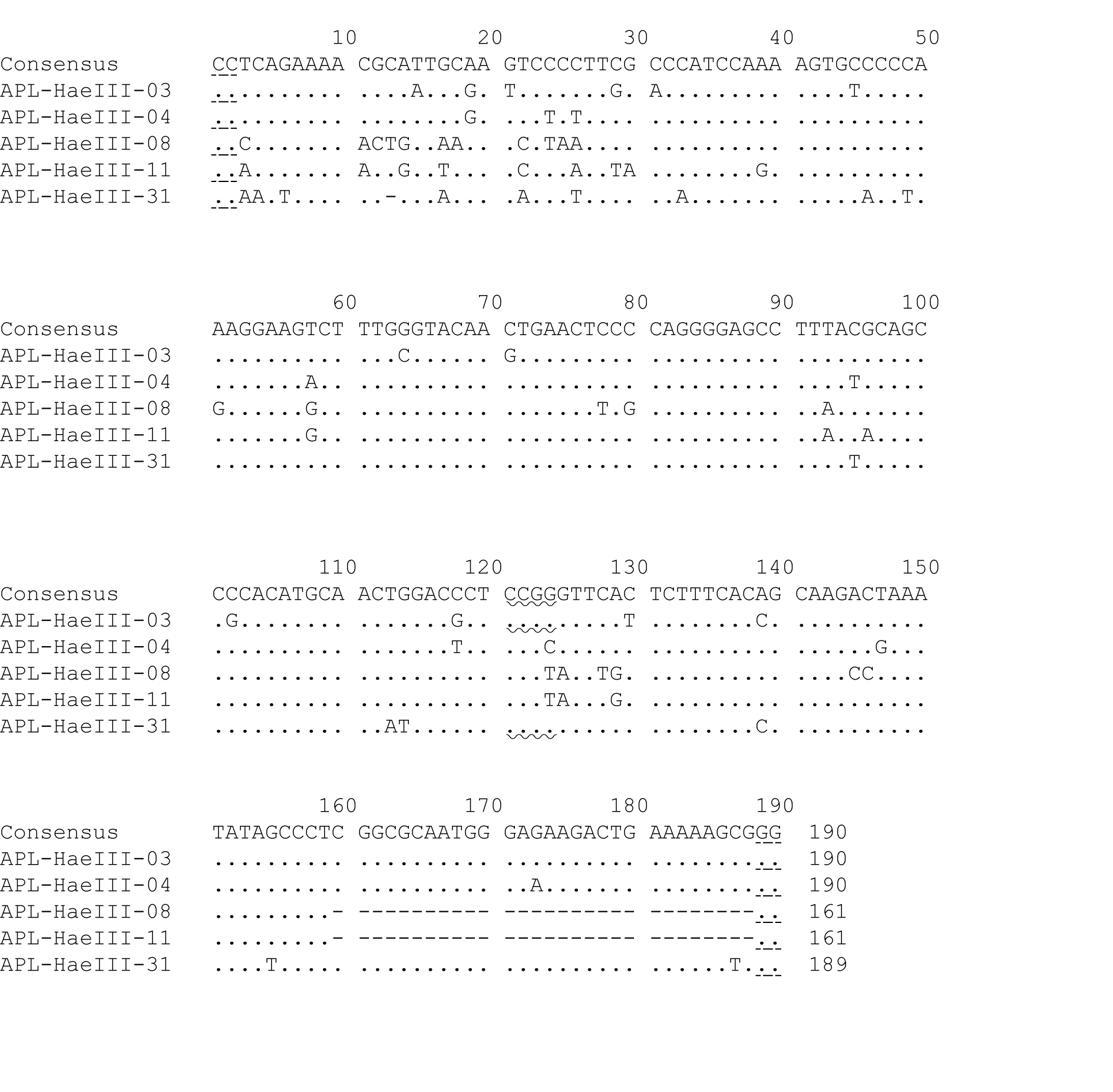

Supplement: S1 Fig — Internal restriction sites of endonucleases are represented by the following underlining: HaeIII, dots and dashes; and MspI, wave. Dots indicate the same nucleotides as those of the consensus sequence shown at the top, and hyphens indicate gaps. (TIF) [file pone.0214028.s001.tif]

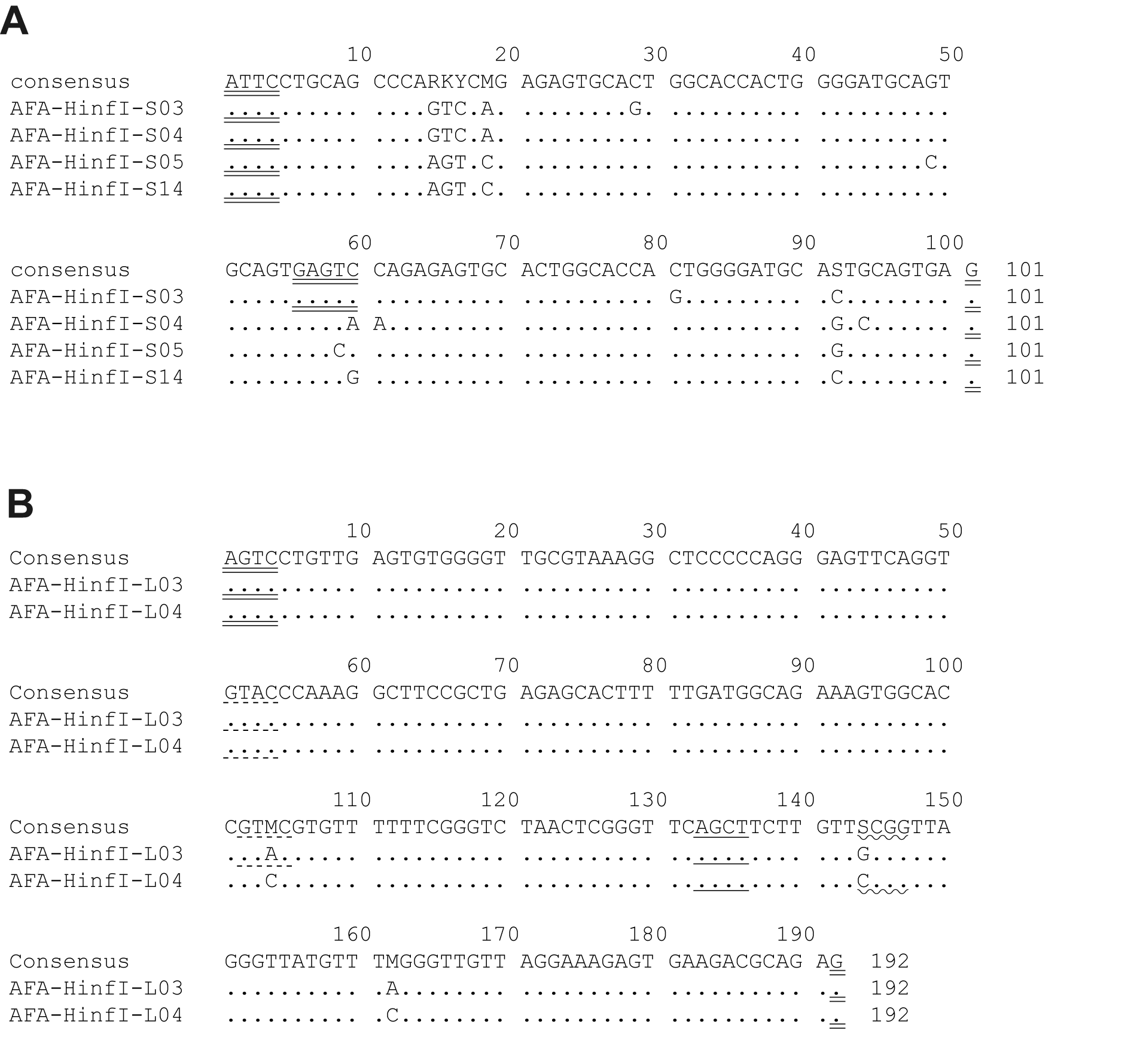

Supplement: S2 Fig — Alignments of nucleotide sequences of four AFA-HinfI-S fragments (A) and two AFA-HinfI-L fragments (B) isolated from the HinfI-digested genomic DNA of A. fabalis. Internal restriction sites of endonucleases are represented by the following underlining: AluI, conventional; HinfI, double; MspI, wave; and RsaI, dots. Dots indicate the same nucleotides as those of the consensus sequence shown at the top, and hyphens indicate gaps. (TIF) [file pone.0214028.s002.tif]

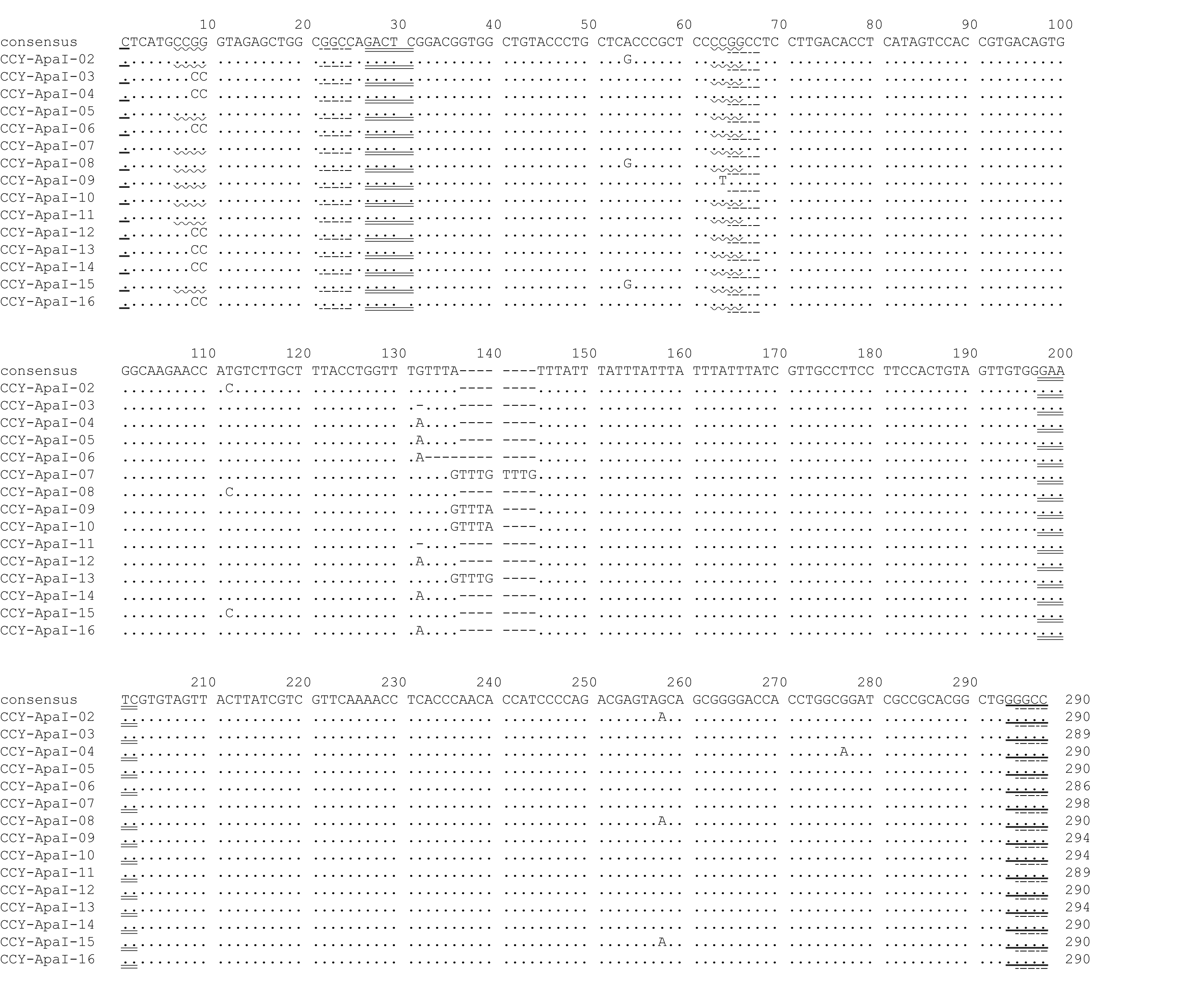

Supplement: S3 Fig — Internal restriction sites of endonucleases are represented by the following underlining: ApaI, bold; HaeIII, dots and dashes; HinfI, double; and MspI, wave. Dots indicate the same nucleotides as those of the consensus sequence shown at the top, and hyphens indicate gaps. (TIF) [file pone.0214028.s003.tif]
